# Gender-Related Factors Influence the Subjective Perception of Deformity in Patients Undergoing Surgery for Idiopathic Scoliosis

**DOI:** 10.3390/jpm13111585

**Published:** 2023-11-09

**Authors:** Davide Bizzoca, Giuseppe Solarino, Anna Maria Moretti, Lorenzo Moretti, Pasquale Dramisino, Andrea Piazzolla, Biagio Moretti

**Affiliations:** 1 UOSD Vertebral Surgery, AOU Consorziale Policlinico di Bari, 70124 Bari, Italydottpiazzola@gmail.com (A.P.); 2 PhD Course in Public Health, Clinical Medicine and Oncology, Department DiMePre-J, University of Bari “Aldo Moro”, 70124 Bari, Italy; 3 Orthopedics Unit, Department of Translational Biomedicine and Neuroscience “DiBraiN”, School of Medicine and Surgery, University of Bari, General Hospital, 70124 Bari, Italy; 4 Department of Pneumology, Santa Maria Hospital, Via De Ferrariis 18/D, 70124 Bari, Italy

**Keywords:** adolescent idiopathic scoliosis, quality-of-life profile for spinal deformities (QLPSDs), spinal appearance questionnaire (SAQ), revised Scoliosis Research Society—22 questionnaire (SRS-22R)

## Abstract

The present study aims to depict the importance of gender-related factors in the subjective perception of spine deformity in adolescents undergoing posterior instrumented fusion for scoliosis. Patients undergoing posterior spinal instrumentation and fusion (PSF) for idiopathic adolescent scoliosis (AIS) were recruited. The following data were recorded: gender, age, parents’ civil status, Tegner Activity Scale (TAS), body mass index (BMI), concomitant diseases, and history of neuropsychological disorders. Each patient underwent clinical and radiological evaluations according to the protocol used at our institution. All the patients were assessed before surgery using the following Patient-Reported Outcome Measures (PROMs): the Italian version of the revised Scoliosis Research Society—22 patient questionnaire (SRS-22R), the Quality-of-Life Profile for Spinal Deformities (QLPSDs) questionnaire, and the Spinal Appearance Questionnaire (SAQ). The present study recruited 80 patients (male: 19, female: 61). A significant correlation was observed between BMI, TAS, and subjective perception scores. A worse deformity perception was observed in female patients and patients with divorced parents. Gender-related factors impact the subjective perception of spine deformity in patients undergoing PSF for AIS. Specific assessment and correction are needed to improve postoperative outcomes in these patients.

## 1. Introduction

Scoliosis is a three-dimensional spine deformity defined by a Cobb angle greater than 10° with combined vertebral rotation [1].

From an etiologic point of view, congenital secondary and idiopathic forms are described [2]. Adolescent Idiopathic Scoliosis (AIS) accounts for more than 80% of daily clinical practice [2].

AIS is a spinal deformity affecting adolescents and poses a significant healthcare challenge due to its unpredictable progression and potential for severe spinal deformity [2]. It typically occurs between the ages of 10 and 18, with no identifiable cause or underlying pathology [2,3]. This condition affects approximately 2–4% of adolescents, with a higher prevalence in females than males [1,2,3].

Despite extensive research, the exact etiology of idiopathic adolescent scoliosis remains elusive [1]. However, multiple genetic, hormonal, and environmental factors have been implicated. Genetic studies have identified certain gene variants associated with scoliosis, suggesting a genetic predisposition [1]. Hormonal imbalances during puberty and abnormal growth patterns are also thought to play a role, although the mechanisms are not fully understood [2].

AIS is most commonly seen in individuals between the ages of 10 and 18, coinciding with rapid growth during puberty [3]. It presents more frequently in females, particularly in those with a family history of scoliosis [3]. The condition varies in severity, with most cases mild, while a small percentage progresses to severe deformities [3].

The early detection and accurate diagnosis of AIS are crucial for effective management. Diagnosis is primarily based on physical examination, including a forward bend test, which demonstrates any asymmetry or rotation of the spine. Radiographic imaging, such as X-rays or MRI, is used to assess curve magnitude and progression and identify any associated anatomical abnormalities [3].

The management of IAS depends on several factors, including the patient’s age, skeletal maturity, and curve severity [1,2,3]. Treatment options include observation, bracing, and surgical intervention [1,2,3]. Observation is usually recommended for mild curves with a low risk for progression [3]. Bracing, such as the Cheneau brace or Milwaukee brace, aims to prevent curve progression by exerting corrective forces on the spine [3,4]. Surgical intervention, such as spinal fusion with instrumentation, is considered for curves exceeding a certain degree of severity or if the condition significantly affects the patient’s quality of life [4].

While most adolescents with AIS can lead fulfilling lives without significant health issues, those with severe curves may experience chronic back pain, respiratory complications, and decreased lung function [3,4]. Regular follow-up and long-term monitoring are essential to identify potential complications and initiate appropriate interventions [3].

Sex- and gender-related factors impact health status throughout the lifespan and influence disease pathogenesis, the response to drug assumption, surgical treatment, and clinical outcome [3].

Sex indicates biological differences between males and females, mainly including hormone levels and their cyclic variation, reproductive/sexual anatomy, and gene expression, which all involve different physiologic and anatomic features [3,4].

Gender includes a complex and multidimensional concept involving several non-biological factors, including sociocultural differences, education level, economic status, psychological aspects, lifestyle, comorbidities, drug assumption, and religious beliefs [3,4].

In recent years, the rapid growth of gender-specific medicine in clinical research [5,6,7,8,9,10], together with the rising importance of the Patient-Reported Outcome Measures (PROMs)—including body image perception—in the assessment of patient satisfaction [5,6,7,8,9,10], has shed new light on the clinical and psychological evaluation of patients with spinal deformity.

The present study aims to depict the importance of gender-related factors in the subjective perception of spine deformity in adolescents undergoing posterior instrumented fusion for AIS.

## 2. Materials and Methods

Patients undergoing posterior spinal instrumentation and fusion (PSF) for idiopathic adolescent scoliosis (AIS) between January 2015 and December 2019 at our Spinal Deformity Center were recruited.

Inclusion criteria: gender: male/female; scoliosis with a Cobb angle ≥40° and a document curve evolution after skeletal maturity; Risser ≥4.

Exclusion criteria: congenital malformations; syndromic scoliosis; neuromuscular scoliosis.

According to the 1964 Declaration of Helsinki, our center’s clinical research ethics provided ethical clearance to the present study protocol (Code: 6479). All the patients and their parents gave their written informed consent before enrolment in the study.

The following data were recorded: gender, age, parents’ civil status, Tegner Activity Scale, body mass index (BMI), number of brothers/sisters, concomitant diseases, and history of neuropsychological disorders.

Each patient underwent clinical and radiological assessment according to the protocol used at our institution. All the patients, before surgery, underwent full-spine upstanding and supine anteroposterior X-rays, lateral upstanding full-spine X-rays, bending X-rays, and Teschendorf X-rays. A full-spine and head 1.5T MRI were also performed to rule out any neurological malformation.

All the patients underwent posterior spinal fusion (PSF) with direct vertebral derotation using a system of titanium screws and rods. At each instrumented level, bilateral pedicle screw insertion was performed; the free-hand technique was used to implant all the pedicles. Facetectomies were performed at all instrumented vertebrae to increase the curve correction. Posterior spinal fusion was achieved by performing laminar decortication and autologous bone graft, deriving from decorticated laminae, spinous processes, and facet joints, at each instrumented vertebra. In all patients, an intradermic suture was performed.

The following Patient-Reported Outcome Measures (PROMs) were used to assess all the recruited patients: the Quality-of-Life Profile for Spinal Deformities (QLPSDs)questionnaire, the Italian version of the revised Scoliosis Research Society—22 patient questionnaire (SRS-22R), and the Spinal Appearance Questionnaire (SAQ).

The Scoliosis Research Society—22R (SRS-22R) questionnaire consists of 22 items belonging to five domains, i.e., function, pain, self-image, mental health, and satisfaction with management [11].

The Quality-of-Life Profile for Spine Deformities (QLPSDs) contains 21 items grouped into five dimensions: psychosocial functioning, sleep disturbances, back pain, body image, and back flexibility [12]. The QLPSD total score ranges from 21 (i.e., the best quality of life) to 105 (i.e., the poorest quality of life) [12].

The Spinal Appearance Questionnaire (SAQ) aims to measure the patient’s perception of spinal deformity [13]. It is composed of 11 items containing standardized drawings showing the varying severity of several components of spinal deformity (SAQ Appearance), followed by 22 questions referring to patients’ impressions regarding their appearance (SAQ Expectation). The SAQ has a total possible score ranging from 14 (best score) to 70 (worst score) [13].

Statistical analysis was performed using SPSS^®^ (version 23; IBM Corp, Armonk, NY, USA). The Shapiro–Wilk test was conducted to verify the normal distribution of the data. Pearson’s correlation test was performed to assess any relationship between the gender-related factors and the SAQ Appearance mean score, SRS-22R domains mean scores, and QLPSDs domains mean scores in Group A patients.

The data are presented in terms of mean value and standard deviation (SD); a *p*-value < 0.05 was considered significant.

## 3. Results

The main data of the study are summarized in Table 1. In total, 80 patients (average age 16.45, range 13–19 years old) were recruited. The biological data (age, sex), the scoliosis curve features (Lenke classification, main curve Cobb angle), the gender-related factors investigated in the present study (BMI, parents’ civil status, TAS, history of neuropsychological disorders), and mean PROMs scores have been reported.

Table 2 shows the Pearson correlation test between gender-related factors and SRS-22 Self-Image mean score, QLPSDs Body Image mean score, and SAQ Appearance mean score. Patients with a lower BMI showed worse SRS-22R Self-Image, QLPSDs Body Image, and SAQ Appearance mean scores. In patients who performed a reduced physical activity (Tegner Activity Scale) or were only children, worse mean SRS-22R Self-Image, QLPSDs Body Image, and SAQ Appearance mean scores were observed.

**Figure 1:**
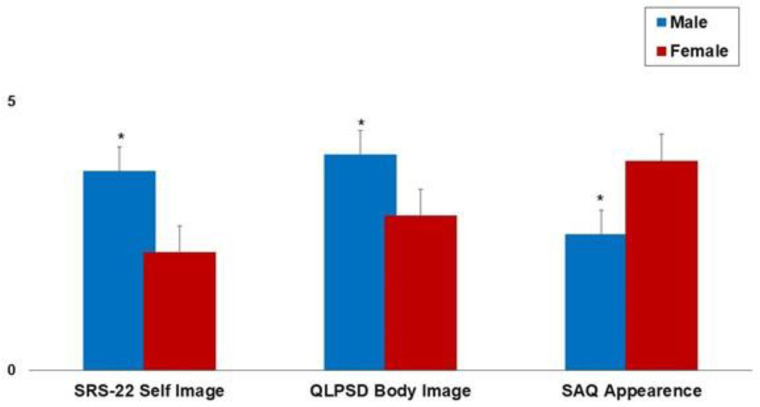
The mean SRS-22 Self Image, QLPSDs Body Image, and SAQ Appearance values in male versus female patients. The error bars indicate the standard deviation. * *p* < 0.05 (Paired *T*-test).

Figure 1 shows the mean SRS-22 Self-Image, QLPSDs Body Image, and SAQ Appearance values in male versus female patients. Better mean scores were observed in male patients compared with female patients. These findings highlight the importance of sex-related factors in the processing of the subjective perception of the deformity.

**Figure 2:**
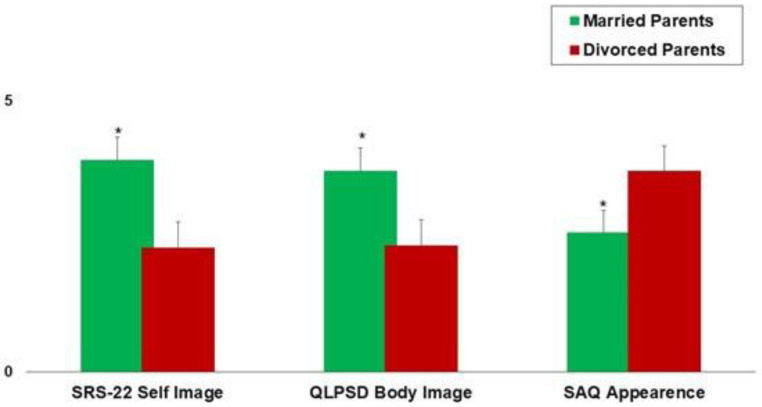
The mean SRS-22 Self Image, QLPSDs Body Image, and SAQ Appearance values in patients with married parents compared to patients with divorce patients. The error bars indicate the standard deviation. * *p* < 0.05 (Paired *T*-test).

Figure 2 shows the mean SRS-22 Self Image, QLPSDs Body Image, and SAQ Appearance values in patients with married parents compared to patients with divorce patients. Better scores have been observed in patients with married parents.

## 4. Discussion

Idiopathic adolescent scoliosis is a tridimensional deformity of the spine [14]. In severe cases, when non-surgical interventions fail to provide adequate correction/curve stabilization, posterior spine fusion surgery is often recommended for adolescents. This procedure aims to stabilize the spine, correct deformities, and improve overall function [14,15,16,17].

Before surgery, a thorough preoperative evaluation is conducted to assess the severity of scoliosis, overall health status, and neurological function of the patient [15]. Common evaluation methods include physical examination, X-rays, and MRI scans. This evaluation helps in determining the appropriate surgical approach and predicting potential risks and complications [1,2,16].

Posterior spine fusion typically involves the use of metal screws, rods, and bone grafts to realign and stabilize the spine. The surgical technique varies depending on the curve magnitude, flexibility, and patient-specific factors [17]. During surgery, the surgeon corrects the curvature and applies hardware to maintain alignment [2,14]. Bone grafts are then placed to promote spinal fusion, which is the union of vertebrae into a solid bone [3,17].

Following surgery, patients are closely monitored in a specialized postoperative unit; the intensive care unit is needed in selected more complex cases [2,15]. Pain management, wound care, and early mobilization are essential during this period [3,15]. The use of intraoperative neuromonitoring techniques helps in detecting any potential nerve injuries and allows for prompt intervention [14,15,16].

Postoperative X-rays are crucial for assessing the correction achieved by the surgery; the Cobb angle is used to quantify the degree of scoliosis [2,17].

The primary goal of posterior spine fusion is to correct and or stabilize the deformity and, in the meantime, improve function and quality of life for adolescents with idiopathic scoliosis [10,14,15,16,17]. Functional outcomes are assessed using various validated outcome measures, including the SRS-22 and the QLPSDs questionnaire. These tools evaluate pain levels, physical and psychological well-being, and overall satisfaction with the surgical outcome [1,10,14,15].

While posterior spine fusion is generally safe and effective, it is not without risks [1,17]. Potential complications include infections, blood loss, nerve damage, implant failure, and nonunion of the fused vertebrae [2,16,17].

Regular long-term follow-up is necessary to monitor postoperative outcomes and assess the stability of the spinal fusion [1,10,14,15,16]. This provides an opportunity to detect potential complications and intervene early if necessary [1,17]. Radiological and functional assessments are typically repeated during follow-up visits to ensure durable outcomes and patient satisfaction [2,10,14,15,16,17].

Gender-related issues in idiopathic adolescent scoliosis have been a topic of interest and concern for healthcare professionals and researchers alike [3,4,5,18,19,20]. Idiopathic scoliosis refers to a tridimensional deformity of the spine, commonly affecting adolescents during their growth spurts [1,14]. While scoliosis impacts individuals of both genders, there are unique gender-related considerations that need to be addressed in the diagnosis, treatment, and overall management of this condition [2,3,10,14,15,16,17].

One gender-related issue is the discrepancy in the prevalence of scoliosis between males and females [1,14]. It has been observed that adolescent girls are more likely to develop scoliosis compared to boys [1,2]. This difference in prevalence raises questions about potential hormonal and genetic factors that could contribute to the development of scoliosis [1,14,15,16]. Understanding these gender-specific risk factors could aid in early detection and targeted intervention, improving outcomes for affected individuals [1,17].

Another issue is the impact of scoliosis on body image and self-esteem, particularly in adolescent girls [1,14,15,16]. The visible physical changes associated with a curved spine can lead to feelings of self-consciousness, resulting in body image dissatisfaction and a negative impact on psychosocial well-being [1,14,15,16]. Adolescent girls, in particular, may face additional societal pressures related to body appearance, exacerbating these issues [14,15,16]. Healthcare providers must be sensitive to these concerns and provide appropriate support and counselling to address the psychological impact of scoliosis [1].

Treatment options for scoliosis also present gender-related challenges [1,14,15,16,17,20]. Bracing and surgical interventions, two mainstay approaches for managing scoliosis, may have different implications for males and females [1,20,21]. For instance, girls with scoliosis may risk developing breast asymmetry due to pressure from their braces or surgical implants [1,10,14,15,16,17]. This potential side effect introduces considerations for physical appearance and self-esteem during the treatment process [1]. In contrast, males may also face unique challenges, such as difficulties fitting and concealing a brace due to differences in body shape [1,10,14,15,16,17]. Identifying and addressing these gender-specific concerns can enhance treatment outcomes and patient satisfaction [20].

Moreover, the impact of scoliosis on physical activity and participation in sports may vary between genders. In some cases, scoliosis can limit flexibility, strength, and range of motion, which may affect participation in certain activities [10,14,15,16,17]. It is important to recognize that adolescent boys and girls may have different sporting interests and expectations, and scoliosis may impact their ability to engage in these activities differently [1]. Healthcare practitioners should work with patients to develop personalized plans that consider their gender preferences and help them maintain an active lifestyle while managing their condition [1,22,23].

Lastly, gender-related differences may also influence access to healthcare and treatment outcomes [1,10,14,15,16,17,19,20,21]. Cultural and societal factors can shape perceptions and stigmas associated with scoliosis, which may impact help-seeking behaviours and access to appropriate care [1,19,20,21,22]. It is crucial to address these disparities and ensure that individuals of all genders have equal access to timely and effective interventions for scoliosis [19,20]. AIS needs surgical treatment when the main curve Cobb angle exceeds 45° since such a kind of deformity is very likely to worsen, even after body growth ends [23]. The surgical treatment of AIS aims at three-dimensional deformity correction, achieving a solid arthrodesis and preventing curve progression in future [1]. All these goals could be achieved with posterior spinal instrumentation and fusion surgery (PSF) with pedicle screws, which currently is the gold standard for the surgical correction of AIS [1].

Severe AIS may have a relevant impact on the patient’s physical appearance since it can lead to a visible trunk deformity, thus predisposing to psychological disturbances [6,7,8].

Body image perception plays a crucial role in the psychological and emotional well-being of adolescents, especially those undergoing surgery for idiopathic scoliosis [6,7,8]. While surgical intervention is often necessary to correct spinal deformity, it can have a profound impact on body image perception in these young individuals [6,7,8]. The surgical procedure itself, involving the placement of rods and screws to straighten the spine, can result in visible changes to the physical appearance [6]. As a result, adolescents may experience feelings of self-consciousness, anxiety, and a negative perception of their bodies [7,8].

The impact of scoliosis surgery on body image perception extends beyond the physical changes [7]. Adolescence is a time of heightened self-awareness and a desire for acceptance and conformity among peers [6]. Individuals undergoing scoliosis surgery may have concerns about having a visible scar, restricted mobility, or differences in posture. These concerns can lead to feelings of social isolation, reduced self-confidence, and even depression [6,7,8]. Moreover, societal pressures and unrealistic beauty standards can further exacerbate these negative body image perceptions.

It is important to address body image concerns in adolescents undergoing scoliosis surgery to promote their overall well-being and mental health [6,7,8]. Healthcare providers and support networks play a significant role in providing appropriate guidance and support to help these individuals develop a positive body image [6]. Psychotherapy and counselling can help adolescents navigate through the emotional challenges associated with body image changes, promoting self-acceptance and resilience [7]. Encouraging open communication, fostering peer support groups, and educating adolescents about the realities of body image can also contribute to a healthy body image perception [8].

Additionally, it is crucial to emphasize the importance of self-identity beyond physical appearance [6]. Adolescents should be encouraged to focus on their strengths, talents, and personal achievements rather than placing excessive importance on physical attributes. Promoting a culture of body positivity and inclusivity can help shift societal perspectives and reduce the stigma surrounding physical differences [6,7,8].

Hence, it has been reported that in patients with AIS, self-perceived body image could be negatively affected by the severity of the deformity, thus ultimately affecting the adolescents’ quality of life [9,10].

Furthermore, Essex et al., in a recent narrative review including fifteen papers, reported that AIS can influence both self-image and appearance perception [10]. The diagnosis of AIS and its subsequent treatment should also be considered a major event and is often accompanied by uncertainty, shock, and anxiety [10].

The present study has analyzed the impact of gender-related factors on the subjective perception of spine deformity in adolescents undergoing posterior instrumented fusion (PSF) for AIS. Eighty adolescents were included in the present study. Subjective image perception has been assessed using the following scores: the SRS-22 Self Image mean score, the QLPSDs Body Image mean score, and the SAQ Appearance mean score.

Female gender, normal–low BMI, and higher TAS mean scores were associated with a worse subjective perception of the body image (Table 2, Figure 2). Patients’ age, on the other hand, showed no correlation with the clinical scores. This finding could be explained by considering the homogenous age of the recruited subjects since only adolescents were included in the present study.

Moreover, parents’ civil status and the number of brothers and/or sisters had a significant impact on the patients’ subjective image perception (Table 2, Figure 2) since worse scores were observed in only-child patients and patients with divorced parents. These findings play a key role in the management of scoliosis since the conservative and surgical treatment of scoliosis involves the cooperation of all family members; thus, differences in the family composition and familial dynamics significantly affect the quality of life and clinical outcomes in adolescents affected by scoliosis.

The data shown in the present study could be useful in defining a personalized treatment protocol for patients suffering from AIS, thus improving the psychological and clinical management of such a kind of patient. These findings could be useful in preoperatively detecting patients who could develop postoperative dissatisfaction, thus helping clinicians in preventing a delayed postoperative recovery. Hence, patients with a higher risk of delayed recovery should undergo a psychological evaluation to avoid postoperative complications.

Moreover, the present findings may guide the surgeon in the surgical planning of patients who could undergo selective posterior spinal fusions: in patients who preoperatively show a negative subjective perception of their body image, non-selective fusions may be recommended to avoid postoperative patient dissatisfaction. In such a kind of patient, therefore, a non-selective posterior vertebral fusion has the advantage of obtaining a higher surgical correction, thus generally providing a better postoperative trunk aesthetic.

The limitations of the present study, however, could not be overcome. First, there Is a lack of follow-up during scoliosis treatment. Moreover, only patients undergoing surgery for scoliosis have been included in the present study. Finally, because of the lack of power analysis, the study sample could not fully represent the real-world population.

## 5. Conclusions

Gender-related factors impact the subjective perception of spine deformity in patients undergoing PSF for AIS. Specific assessment and correction are needed to improve postoperative outcomes in these patients.

## Figures and Tables

**Table 1:** Main data of the study.

Patients (*n*)	80
**Age**	
Mean ± SD	16.45 ± 2.12
Range	13–19
**Gender**	
Male, n (%)	19 (23.75%)
Female, n (%)	61 (76.25%)
**BMI (Kg/m^2^)**	
Mean ± SD	21.64 ± 1.77
**Parents’ civil status**	
Married, n (%)	69 (86.25%)
Divorced, n (%)	11 (13.75%)
**Tagner Activity Level Scale (TAS)**	
Mean ± SD	5.4 ± 2.1
**History of neuropsychological disease**	
n (%)	2 (2.5%)
**Main curve Cobb**	63.3 ± 12.88
**Secondary curve Cobb**	32.65 ± 10.57
**Lenke Classification**	
Lenke 1, n (%)	38 (47.5%)
Lenke 2, n (%)	22 (27.5%)
Lenke 3, n (%)	7 (8.75%)
Lenke 4, n (%)	7 (8.75%)
Lenke 5, n (%)	4 (5%)
Lenke 6, n (%)	2 (2.5%)
**SRS-22R Self-Image**	
Mean ± SD	2.05 ± 0.67
**QLPSDs Body Image**	
Mean ± SD	2.34 ± 0.55
**SAQ Appearance**	
Mean ± SD	2.27 ± 0.46

**Table 2:** Pearson correlation test between gender factors and SRS-22 Self-Image mean score, QLPSDs Body Image mean score, and SAQ Appearance mean score.

Gender-Related Factors	SRS-22R Self-Image		QLPSDs Body Image		SAQ Appearance	
	R	*p*	R	*p*	R	*p*
Age	0.322	0.32	0.24	0.112	0.221	0.21
BMI	0.845	0.001 *	0.622	0.01 *	−0.677	0.03 *
Tagner Activity Scale	0.693	0.008 *	0.733	0.003 *	−0.684	0.02 *
Number of brothers/sisters	0.816	0.002 *	0.678	0.01 *	−0.711	0.008 *

* Significant *p*-value.

## Data Availability

The data that support the findings of this study are available from the corresponding author, upon reasonable request.
